# Varicocoele embolization with sclerosing agents leads to lower radiation exposure and procedural costs than coils: Data from a real‐life before and after study

**DOI:** 10.1111/andr.13162

**Published:** 2022-02-26

**Authors:** Luca Boeri, Irene Fulgheri, Marco Cristina, Pierpaolo Biondetti, Silvia Rossi, Elena Grimaldi, Gianpaolo Lucignani, Franco Gadda, Anna Maria Ierardi, Andrea Salonia, Paola Viganò, Edgardo Somigliana, Gianpaolo Carrafiello, Emanuele Montanari

**Affiliations:** ^1^ Department of Urology Foundation IRCCS Ca’ Granda Ospedale Maggiore Policlinico Milan Italy; ^2^ Università degli Studi di Milano Milan Italy; ^3^ Department of Vascular Surgery Foundation IRCCS Ca’ Granda Ospedale Maggiore Policlinico Milan Italy; ^4^ Department of Radiology Foundation IRCCS Ca’ Granda Ospedale Maggiore Policlinico Milan Italy; ^5^ Division of Experimental Oncology/Unit of Urology URI IRCCS Ospedale San Raffaele University Vita‐Salute San Raffaele Milan Italy; ^6^ Infertility Unit Foundation IRCCS Ca’ Granda Ospedale Maggiore Policlinico Milan Italy

**Keywords:** coils, cost, embolization, sclerosing agents, spermatozoa, varicocoele

## Abstract

**Objectives:**

To investigate clinical outcomes, radiation exposure and procedural costs associated with percutaneous varicocoele embolization using coils and sclerosing agents (SAs) in a cohort of young‐adult men.

**Materials and methods:**

Data from consecutive men treated with percutaneous varicocoele embolization using coils and SA between 2017 and 2021 were analyzed. The allocation was based on a change of policy occurred in June 2020 with the substitution of coils with SA (before and after study). Semen analysis values were based on 2010 WHO reference criteria. Anatomic variants of gonadal veins were categorized according to Jargiello et al. Intraoperative radiation dose and procedural costs were collected for each patient. Descriptive statistics and linear regression models were used to describe the association between clinical parameters with procedural costs and radiation exposure.

**Results:**

One hundred sixteen men were included, of whom 76 (65.5%) received coils, and 40 (34.5%) received SA. Baseline characteristics of the two study groups did not differ. A type 3 Jargiello anatomic variation of left gonadal vein was found in 45.7% of cases. Radiation dose was lower in the SA group as compared to the coils one (13.2 [7–43] vs. 19.8 [12–57] Gy/cm^2^; *p* < 0.001). Similarly, procedural costs were lower for the SA group (169.6 [169–199] € vs. 642.5 [561–775] €; *p* < 0.001). At follow‐up, pain and sperm variables significantly improved in both groups (*p* < 0.01), without differences among the embolic materials. Linear regression model revealed that coils use was associated with higher radiation exposure (beta 8.8, *p* = 0.02) than SA after accounting for anatomic variation of gonadal vein, body mass index, and vascular access.

**Conclusions:**

SA and coils for varicocoele embolization are equally safe and effective. The use of SA was associated with lower radiation exposure and procedural costs than coils. These results should be considered in terms of public health cost and patient's safety.

## 1 INTRODUCTION

Varicocoele is a common congenital abnormality that may be associated with several andrological conditions, including couple's infertility, reduced testicular volume, testicular pain, and hypogonadism.^1^ It is estimated that varicocoele may occur in almost 15% of the general male population and in 40% of men presenting for couple's infertility.^2^, ^3^ The exact pathophysiological mechanism underlying the association between varicocoele and male subfertility is still debated, but increased scrotal temperature, hypoxia, and reflux of toxic metabolites causing testicular dysfunction and deoxyribonucleic acid damage are the leading hypotheses.^2^

Treatment of varicocoele is indicated in symptomatic patients, in adolescents with ipsilateral reduction of testicular volume and progressive testicular dysfunction and in infertile men with clinical varicocoele, abnormal semen parameters, and healthy female counterpart.^3^ Several techniques of varicocoele repair have been proposed including open surgical, laparoscopic, microsurgical, and percutaneous radiological approaches, all of which are associated with different rates of recurrence and complications.^4^, ^5^ In adults and pediatric population, percutaneous embolization of varicocoele has widely spread in clinical practice due to its minimally invasive nature and high success rates (approximately 90%).^6^

Coils and sclerosing agents (SAs) are among the most commonly used embolic materials during percutaneous embolization of varicocoele. A recent systematic review analyzed data from 3505 subjects treated with varicocoele embolization using coils, glue, and SA.^7^ Authors found a 90% success rate after surgery, which was independent from the embolic material. However, mechanical embolization with coils resulted in slightly higher recurrence rates (8%–11%) in the long term follow‐up.^7^

Of clinical importance, few studies have investigated procedural costs and radiation exposure associated with different embolic materials during varicocoele treatment. These aspects are critically relevant in terms of public health costs and patient's safety in light of the known negative impact of radiations on reproductive organs.

Therefore, in this study we sought to investigate (i) clinical outcomes, (ii) radiation exposure, and (iii) procedural costs associated with varicocoele embolization using coils and SA in a cohort of young adult men.

## 2 METHODS

We performed a retrospective analysis of data prospectively collected from young adult patients assessed for varicocoele at a single academic center between January 2017 and March 2021.

All participants were assessed with a thorough medical history. The Charlson Comorbidity Index was used to score health‐significant comorbidities, coded using the International Classification of Diseases, 10th revision.^8^, ^9^ Likewise, weight and height were measured, calculating body mass index (BMI) for each participant. Testes volume was assessed in all cases using Prader's orchidometer estimation.^10^ Smoking habit was investigated according to the pack‐year history and then categorized into two groups, as follows: no smokers/former smokers and active smokers. Colour‐Doppler ultrasound (CDUS) was used to detect spermatic vein reflux and to classify the grade of varicocoele in every subject, according to the Sarteschi classification.^11^

Semen samples were collected by masturbation and analyzed within 2 h according to the WHO criteria.^12^ For the specific purposes of this study, we evaluated sperm volume, sperm concentration, progressive motility, and normal morphology.

### 2.1 Procedure details

All procedures were performed in outpatient setting by one of the six institutional interventional radiologists. After performing local anesthesia (lidocaine 2%), a right femoral vein access was gained with ultrasound guidance using the Seldinger technique, and a 5F sheath was placed. Jugular access could also be considered, according to the operator preference. Under fluoroscopic guidance, a J‐shaped 0.035 inch hydrophilic guide wire and a 5F catheter were used to selectively catheterize the left renal and then internal spermatic vein; depending on the operator preference, a cobra (Terumo, Tokyo, japan or Cordis, Miami, FL) or a renal double curve (Cordis, Miami, FL) catheters were used. Fluoroscopic study of the left renal and the internal spermatic veins was performed in order to assess venous anatomy and reflux degree. Contrast injection was done with the patient performing Valsalva manoeuvre. In case of presence of more than one vein, the aim was to embolize each vessel draining the pampiniform plexus.

Embolization was performed at the level of the distal segment of the spermatic vein either mechanically, using 0.038 coils, or with polidocanol (Atoxysclerol) at 3% (namely, SA). Before injection, liquid polidocanol was turned into foam through its agitation with air between two syringes through a three‐way tap with a liquid‐air ratio of 1:3; we used a dose of 1 mg/kg up to a limit of 80 mg. Foam was injected by hand with the patient performing Valsalva maneuver, to reduce reflux, and, at the same time, either manually compressing the inguinal ring or repeatedly touching the left testicle, to avoid penetration of the sclerosant into the pampiniform plexus.

If possible, digital subtraction angiography was avoided while collimation was used. Fluoroscopy time and mode were kept as low as possible, limiting the use of magnification and avoiding oblique projections.

From 01/2017 to 05/2020, all procedures were performed with coils; conversely, from 06/2020 to 03/2021 SA was used as embolic material. This situation allowed us to design a before and after study.

**FIGURE 1:**
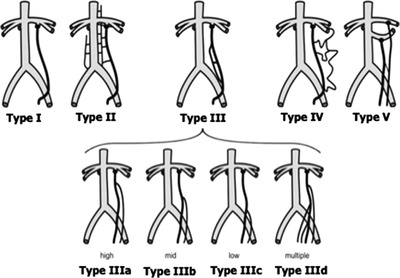
Anatomic variants of left gonadal vein

Anatomic variants of gonadal veins were categorized according to the classification proposed by Jargiello et al^13^ (Figure 1). Two experienced radiologists (SR; EG), blinded to each other, reviewed all intraoperative images for scoring gonadal vein anatomy. Cases of interobserver disagreements were resolved by a third party (PB).

Patients were discharged after few hours of clinical observation, and they returned to their normal daily activities after 24–48 h.

All subjects were instructed to access the hospital emergency department in case they developed postsurgical complications. Follow‐up, office‐based visits were routinely scheduled 6 months after surgery as per standard clinical protocol, and patients were asked to repeat a semen analysis. At follow‐up visit, all patients underwent a CDUS.

Radiation exposure during the embolization procedure was assessed by the dose area product (DAP, Gy/cm^2^). The accounts department of the hospital provided detailed expense costs, which were compared among procedures. In particular, we recorded the cost of interventional and embolic materials.

Data collection followed the principles outlined in the Declaration of Helsinki. Since retrospective, a specific informed consent was not foreseen. However, all patients signed an informed consent form for their data to be used for research purposes. The study was approved by the local ethical committee.

### 2.2 Statistical methods

Distribution was tested with the Shapiro–Wilk test. Data are presented as medians (interquartile range [IQR]) or frequencies (proportions). First, demographic characteristics were compared between coils and SA groups with the Mann–Whitney test and the chi‐square test. Second, the paired *t*‐test assessed potential differences in sperm parameters at follow‐up assessment compared to baseline among both groups. Sperm variables were also compared between surgeries at follow‐up with the Mann–Whitney test. Finally, univariable and multivariable linear regression analyses tested the associations between study variables and radiation dose. Statistical analyses were performed using SPSS v.26 (IBM Corp., Armonk, NY, USA). All tests were two sided, and statistical significance level was determined at *p* < 0.05.

## 3 RESULTS

One hundred thirty‐five subjects were initially selected. We then excluded those lost to follow‐up (*n* = 3; 2.2%), those treated for bilateral varicocoele (*n* = 1; 0.7%), those with a normal phlebogram (*n* = 7; 5.2%), and those who could not be treated because of a technical failure (*n* = 12; 8.8%). A final cohort of 116 (85.9%) men submitted to left‐sided varicocoele embolization was considered for statistical analysis.

Table 1 details demographic characteristics of the whole cohort. Overall, median (IQR) patient's age and BMI were 30 (24–33) years and 25.5 (23.7–29.4) kg/m^2^, respectively. Indication for varicocoele treatment was scrotal pain and couple's infertility in 44 (37.9%) and 57 (49.1%) cases, respectively. Of 116, 66 (56.9%) men had CDUS grade ≥4 varicocoele. SA and coils were used in 40 (34.5%) and 76 (65.5%) patients, respectively, with a jugular access in 66 (56.9%) cases. The Jargiello type III was the most frequent anatomic variation of the left gonadal vein (*n* = 53; 45.7%). Median preoperative sperm concentration, total motility, and normal morphology were 15.0 (5.5–25) x10^6^/ml, 35 (25–40) %, and 3 (2–6)%, respectively. The overall technical success rate was 97%. In the whole cohort, median DAP and procedural costs were 18.6 (11–47) Gy/cm^2^ and 489.3 (196.5–705.2) €, respectively.

**TABLE 1:** Demographic characteristics of the whole cohort of patients (number = 116)

Age (years)	
Median (IQR)	30.0 (24–33)
Range	20–42
BMI (kg/m^2^)	
Median (IQR)	25.5 (23.7–29.4)
Range	19.9–41.0
CCI (value)	
Median (IQR)	0.0 (0.0)
Mean (SD)	0.9 (0.4)
Range	0–3
Smoking status (number [%])	
No/former smokers	73 (62.9)
Current smokers	43 (37.1)
Left testis volume (Prader estimation)	
Median (IQR)	18.0 (15–20)
Range	10–25
Clinical indication (number [%])	
Pain	44 (37.9)
Infertility	57 (49.1)
Pain and infertility	15 (13.0)
Varicocoele grade (number [%])	
III	50 (43.1)
IV	47 (40.5)
V	19 (16.4)
Anatomic variant of left gonadal vein (number [%])	
I	37 (31.9)
II	10 (8.6)
III	53 (45.7)
IV	13 (11.3)
V	3 (2.5)
Embolic material (Number [%])	
Coils	76 (65.5)
Sclerosing agent	40 (34.5)
Percutaneous access (Number [%])	
Jugular	66 (56.8)
Femoral	46 (39.6)
Combined	4 (3.6)
Dose area product (Gy/cm^2^)	
Median (IQR)	18.6 (11–47)
Range	1.7–200
Total procedural cost (Euros)	
Median (IQR)	489.3 (196.5–705.2)
Range	169.5–1551.3
**Preoperative semen parameters**	
Semen volume (ml)	
Median (IQR)	3.0 (2–4)
Range	1.1–7.0
Sperm concentration (x10^6^/ml)	
Median (IQR)	15.0 (5.5–25)
Range	0.5–100.0
Progressive motility (%)	
Median (IQR)	35.0 (25–40)
Range	1.0–80.0
Normal morphology (%)	
Median (IQR)	3.0 (2.0–6.0)
Range	0.0–80.0

Abbreviations: BMI, body mass index; CCI, Charlson Comorbidity Index; IQR, interquartile range; SD, standard deviation.

Table 2 details descriptive statistics of the population as segregated according to the embolic material. SA and coils groups were similar in terms of age, BMI, preoperative semen parameters, and varicocoele severity. Radiation dose was lower in the SA group as compared to the coils one (13.2 [7–43] Gy/cm^2^ vs. 19.8 [12–57] Gy/cm^2^; *p* < 0.001). Similarly, procedural costs were lower for the SA group (169.6 [169.5–199.7] € vs. 642.5 [561.3–775] €; *p* < 0.001). Postoperative complications were similar among groups (two patients had epididymo‐orchitis in SA group, and one had hydrocele in the coils group).

**TABLE 2:** Descriptive statistics of the whole cohort of patients as segregated according to the embolic material (number = 116)

	Coils	Sclerosing agent	*p*‐Value*
Number of patients (number [%])	76 (65.6)	40 (34.5)	
Age (years)			0.6
Median (IQR)	30.0 (24–33)	29.0 (23–32)	
Range	20–42	20–39	
BMI (kg/m^2^)			0.4
Median (IQR)	25.4 (23.5–27.8)	25.6 (23.8–28.1)	
Range	19.9–41.0	20.5–40.1	
CCI (value)			0.9
Median (IQR)	0.0 (0.0)	0.0 (0.0)	
Mean (SD)	0.9 (0.4)	0.8 (0.4)	
Range	0–3	0–3	
Smoking status (number [%])			0.2
No/former smokers	47 (61.8)	26 (65.0)	
Current smokers	29 (38.2)	14 (35.0)	
Left testis volume (Prader estimation)			0.8
Median (IQR)	18.0 (15–20)	18.0 (15–20)	
Range	10–25	10–25	
Clinical indication (number [%])			0.1
Pain	28 (36.8)	16 (40)	
Infertility	37 (48.6)	20 (50)	
Pain and infertility	11 (14.6)	4 (10)	
Varicocoele grade (number [%])			0.3
III	33 (43.4)	17 (42.5)	
IV	30 (39.5)	17 (42.5)	
V	13 (17.1)	6 (15)	
Anatomic variant of left gonadal vein (number %])			0.1
I	28 (36.8)	9 (22.5)	
II	6 (7.9)	4 (10)	
III	33 (43.5)	20 (50)	
IV	7 (9.3)	6 (15)	
V	2 (2.5)	1 (2.5)	
Dose area product (Gy/cm^2^)			0.02
Median (IQR)	19.8 (12–57)	13.2 (7.0–43.1)	
Range	2.0–200.0	1.7–164.0	
Total procedural cost (Euros)			<0.001
Median (IQR)	642.6 (561.3–775.0)	169.6 (169.5–199.7)	
Range	417.3–1551.3	169.5–993.7	

Abbreviations: BMI, body mass index; CCI, Charlson Comorbidity Index; IQR, interquartile range; SD, standard deviation.

* *p ‐* Value according to the Mann–Whitney test for continuous data and the chi‐square test for categorical variables, as indicated.

At follow‐up, sperm parameters significantly improved in both groups (all *p* < 0.001), without differences according to the embolic materials (Table 3). Orchialgia relief was reported by 98% of men treated for scrotal pain.

**TABLE 3:** Semen parameters of the whole cohort as segregated according to the embolic material (median [interquartile range, IQR])

	Coils	Sclerosing agents	*p*‐Value*
Semen volume (ml)			
Preoperative	3.0 (2.0–4.0)	3.0 (2.0–5.0)	0.9
6 months	3.0 (2.1–4.1)	3.0 (2.1–4.8)	0.8
Sperm concentration (x10^6^/ml)			
Preoperative	15.0 (5.0–25)	14.0 (5.0–23)	0.3
6 months	20.0 (6.2–28)^§^	20.0 (6.1–28)^§^	0.8
Progressive motility (%)			
Preoperative	34.0 (25–40)	35.0 (25–41)	0.6
6 months	39.0 (26–45)^§^	40.0 (27–47)^§^	0.7
Normal morphology (%)			
Preoperative	3.0 (1–5)	3.0 (2–6)	0.2
6 months	4.0 (2–7)	4.0 (2–6)	0.5

* *p*‐Value according to unpaired Mann–Whitney test.

^§^ *p* < 0.001 versus preoperative. *p*‐Value according to paired *t*‐test.

Among the whole cohort, radiation dose increased with increasing complexity of vein anatomy (*p* < 0.01) and in cases performed by jugular access (*p* = 0.02) (data not shown). Table 4 reports univariable and multivariable linear regression analyses testing the association between clinical predictors and radiation dose. Univariable linear regression model revealed that patient's BMI (beta 1.76; *p* = 0.01), jugular access (beta 5.93; *p* = 0.04), a type IV/V anatomy of gonadal vein (beta 7.81; *p* < 0.01), and coils use as embolic material (beta 8.76; *p* = 0.03) were all associated with higher radiation dose during varicocoele embolization. At multivariable logistic regression analysis, a type IV/V anatomy of gonadal vein (beta 7.83; p < 0.01) and coils use as embolic material (beta 8.82; *p* = 0.02) emerged as independent predictors of higher radiation dose during surgery, after accounting for BMI and jugular access.

**TABLE 4:** Linear regression models predicting radiation exposure in the whole cohort

	UVA model	MVA model
	beta; *p*‐value (95% CI)	beta; *p*‐value (95% CI)
Age	−0.2; 0.5 (−0.91 to 0.52)	
BMI	1.76; 0.01 (1.34–3.84)	1.43; 0.1 (−1.77 to 2.75)
Varicocoele severity		
III versus IV/V grade	2.64; 0.3 (−1.29 to 6.34)	
Jugular access	5.93; 0.04 (2.45–10.34)	2.42; 0.2 (−1.34 to 3.97)
Left gonadal vein anatomy		
I–II	Ref.	Ref.
III	5.67; 0.04 (4.12–10.31)	4.23; 0.07 (−1.54 to 5.98)
IV–V	7.81; <0.01 (5.19–12.43)	7.83; <0.01 (4.12–12.86)
Coils versus sclerosing agent	8.76; 0.03 (2.73–15.32)	8.82; 0.02 (3.12–15.45)

Abbreviations: BMI, body mass index; CI, confidence interval; MVA, multivariate model; UVA, univariate model.

## 4 DISCUSSION

Percutaneous treatment of varicocoele was found to be a safe and effective procedure irrespective of the embolic agent,^7^ but an accurate investigation of costs and radiation exposure associated with different embolic materials is currently lacking.

Here we found that SA and coils were equally effective for the treatment of varicocoele in young adult men; however, treatment with SA emerged to be a cheaper procedure than the one with coils and SA was associated with lower radiation exposure than solid embolic material. Taking together, these findings would suggest that varicocoele embolization with SA could be preferable than coils in terms of public health costs and patient's safety from radiation exposure.

Our study was motivated by the substantial lack of research concerning costs and radiation safety during percutaneous varicocoele embolization with different materials. We took the opportunity of a local policy change in the strategy of embolization to address this aspect. Since the first description in 1978 by Lima et al.,^14^ percutaneous embolization of varicocoele has been widely evolved and introduced in clinical practice, due to its minimally invasive nature and excellent clinical outcomes.^3^, ^6^, ^7^, ^15^ This procedure offers a rapid recovery and can be successfully accomplished in approximately 90% of attempts.^6^ Various studies have demonstrated improvement in semen variables following embolization^15^; moreover, percutaneous embolization is a viable treatment option for men suffering from orchalgia secondary to varicocoele. For instance, Puche–Sanz et al. analyzed data from 154 men treated with varicocoele embolization for scrotal pain and showed that at 39 months follow‐up, 86.9% of patients had complete resolution of symptoms.^16^ Concerning varicocoele recurrence, most of surgical failures result from undiagnosed gonadal vein duplications. One advantage of embolization over surgical ligation is the possibility to perform intra‑operative venography that can identify venous anatomic variants. However, published literature reported higher postembolization recurrence (4%–27%) as compared to microsurgical varicocoelectomy (0%–3%).^2^ Major complications of embolization are rare, and minor complications include epididymitis (3%) and hydrocele (0%–12%).^6^

Different embolic materials have been used in clinical practice for varicocoele embolization. Solid embolics, including coils, exert mechanical occlusion of targeted veins, while liquid embolics (glue and SA) induce an inflammatory reaction, resulting in endothelial necrosis and thrombosis.^17^ The choice of the embolic agent usually depends on operator preference, but there are no solid data showing the superiority of one material over the other.

Bilreiro et al. retrospectively analyzed a series of 129 men treated with varicocoele embolization between 2012 and 2015 at a single center.^18^ Glue and coils were used in 26 (20.2%) and 103 (79.8%) cases, respectively. Authors found that clinical success rates, complications, and recurrence rates were similar among groups, but procedure with glue was faster than those with coils.^18^ Similarly, Favard et al. investigated pain and recurrence rates in 182 patients undergoing varicocoele embolization with three different embolic materials.^19^ They found that procedures with glue, coils, and SA were similar in terms of pain reduction, but the use of glue was associated with lower recurrence rates and shorter operative time compared to the other embolic agents.^19^ Lastly, a recent systematic review, including 23 retrospective and seven prospective clinical studies with a total of 3505 patients, assessed the safety and effectiveness of the various embolic materials used in varicocoeles embolization.^7^ Authors showed a technical success rates above 90% for all embolic materials without any significant differences. The safety profile was similar for each embolic agent, but glue appears to be the most effective in preventing recurrence compared to coils and SA.^7^ Our results are in line with these findings. We showed a 97% success rate regardless of the embolic material used and few postoperative complications in both groups. Moreover, sperm variables significantly improved without differences according to the embolic agent.

Since these procedures are performed under fluoroscopic guidance, patients are at risk of radiation exposure. Radiation exposure is linearly related to an increased risk for secondary malignancy^20^; therefore, all efforts should be made to recognise the radiation risk and minimise exposure of patients and other susceptible individuals.^21^ Very little is known about radiation exposure during varicocoele embolization. Favard et al. analyzed radiation exposure during embolization with glue, coils, and SA.^19^ Authors showed that glue procedures had the shortest fluoroscopy time; moreover, DAP was lower in glue than coils group, but no difference was noted between glue and SA, and between coils and SA.^19^ In the present study, we showed that radiation dose was lower in varicocoele embolization with SA than coils. Additionally, radiation exposure increased with increasing complexity of vein anatomy and in cases performed by jugular access. This difference in radiation dose is likely related to the shorter embolic time, both performed under fluoroscopic guidance, when using SA compared to coils. Furthermore, jugular access is characterized by higher radiation doses than femoral one because of the longer anatomical passage that includes thoracic organs.

In healthcare management, cost effectiveness is a crucial component in the evaluation of any diagnostic or therapeutic intervention. Various cost analyses have been reported in urology/radiology setting^22^, ^23^ but not for varicocoele embolization. Here, we reported that embolization with SA was associated with reduced procedural costs compared to coils, with no impact on clinical outcomes. Therefore, these findings gain important implications from a clinical and economic standpoint.

The clinical implication of our study is several‐fold. First, we conducted the first real‐life investigation of varicocoele embolization with SA and coils in terms of clinical outcomes, radiations exposure, and procedural costs in a cohort of young‐adult men. Second, we revealed for the first time that procedures with SA were associated with lower radiation exposure than those with coils. Radiation to the gonads is associated with an increased risk of infertility or future malignancy, and young patients may be at highest risk given their long‐life expectancy. Therefore, embolization with SA should be preferred for radiation safety. Lastly, procedures with SA were cheaper than those with coils. Overall, because of similarity in clinical outcomes, lower radiation exposure, and costs associated with SA, this embolic agent could be advantageous in terms of public health costs and patient's safety.

Our study is not devoid of limitations. First, this was a single center‐based study, raising the possibility of selection biases; thereof, larger studies are needed to externally validate our findings. Second, the retrospective nature of this study limits the generalization of our results. A randomized controlled trial would have obviously been more informative. In this regard, it must however be underlined that a before and after study design has several advantages, the most relevant being the nonexperimental setting.^24^ Lastly, despite not being the primary aim of this investigation, we lack long‐term follow‐up data to investigate recurrence rates among the two groups.

## 5 CONCLUSIONS

This cross‐sectional, real‐life study showed that varicocoele embolization with SA and coils is a safe and effective procedure with clinical outcomes comparable among the two embolic agents. The use of SA was associated with lower radiation exposure and procedural costs than coils. These results should be considered in terms of public health cost and patient's well‐being in light of the known negative impact of radiation on reproductive organs. Further studies are needed to externally confirm these observations.

## FUNDING INFORMATION

None.

## CONFLICT OF INTEREST

The authors declare that there is no conflict of interest that could be perceived as prejudicing the impartiality of the research reported.

## AUTHOR CONTRIBUTIONS

*Collected data*: Marco Cristina, Pierpaolo Biondetti, Silvia Rossi, Elena Grimaldi, Gianpaolo Lucignani, Luca Boeri, Irene Fulgheri, Franco Gadda, Anna Maria Ierardi, Paola Viganò, Edgardo Somigliana, Gianpaolo Carrafiello and Emanuele Montanari. *Revised the manuscript*: Franco Gadda, Anna Maria Ierardi, Andrea Salonia, Paola Viganò, Edgardo Somigliana, Gianpaolo Carrafiello and Emanuele Montanari. *Supervised the project*: Andrea Salonia, Paola Viganò, Edgardo Somigliana, Gianpaolo Carrafiello and Emanuele Montanari. *Designed the study*: Luca Boeri and Irene Fulgheri. *Wrote the paper*: Luca Boeri.
